# In Memoriam: Richard A. Gatti, MD (1937–2025)

**DOI:** 10.70962/jhi.20250225

**Published:** 2025-12-30

**Authors:** Alessandro Aiuti, Luigi D. Notarangelo

**Affiliations:** 1 Università Vita Salute San Raffaele and San Raffaele Telethon Institute for Gene Therapy, IRCCS Scientific Institute San Raffaele, Milan, Italy; 2 Immune Deficiency Genetics Section and Laboratory of Clinical Immunology and Microbiology, National Institutes of Health, Bethesda, MD, USA

## Abstract

Dr. Richard A. Gatti, Professor Emeritus of Pathology and Laboratory Medicine at UCLA, was a visionary in immunology and genetics whose work reshaped the understanding and treatment of inherited immune diseases. Early in his career, he co-authored a landmark 1968 Lancet paper describing the first successful bone marrow transplant for severe combined immunodeficiency, marking the birth of curative transplantation for primary immunodeficiencies. After joining UCLA in 1974, he dedicated his research to ataxia-telangiectasia (A-T), mapping its gene to chromosome 11q23 and contributing to the discovery of genes responsible for DNA repair defects and cancer susceptibility. He co-founded the UCLA Molecular Diagnostics Laboratory and mentored generations of scientists. Beyond science, Dr. Gatti was admired for his warmth, humor, and musicianship. He will be remembered as a pioneering researcher, compassionate clinician, and devoted advocate for patients and families.

## Teaser

Remembering Richard A. Gatti, MD, a pioneer of clinical immunology and hematopoietic stem cell transplantation, whose landmark contributions—from the first curative bone marrow transplant for primary immunodeficiencies to the discovery of the ATM gene—transformed the diagnosis and treatment of rare immune and DNA repair disorders.

It is with sadness that we commemorate the passing of Dr. Richard A. Gatti, Professor Emeritus, Pathology and Laboratory Medicine, University of California, Los Angeles (UCLA). After obtaining a medical degree at St. Louis University Medical School, Dr. Gatti’s medical career began in pediatrics, enriched by experiences that combined patient care with deep curiosity about the immune system. He completed his residency at Northwestern University in Chicago, then pursued a research fellowship in biochemical genetics at the United States Public Health Service (USPHS). In 1968, he embraced a USPHS Research Fellowship in Immunology at the University of Minnesota under the mentorship of Dr. Robert Good, who is often considered the father of clinical immunology. In 1968, Dr. Gatti, working with Dr. Good and colleagues, co-authored a landmark report in *The Lancet* describing the first successful bone marrow transplantation from a female sibling donor ever performed in humans in a 5-mo-old child with X-linked severe combined immunodeficiency (1). The compatibility of the sibling donor at the HLA locus was determined by mixed lymphocyte reaction and lymphocytotoxic assays. A mild graft-versus-host (GvH) reaction appeared at 8 days after implantation but resolved spontaneously. A progressive and sustained increase in the count of donor-derived peripheral blood lymphocytes and of immunoglobulin serum levels was observed. However, about 3 mo later, bone marrow failure developed. A second transplantation of bone marrow cells from the same donor allowed complete hematological recovery and immune reconstitution without evidence of GvH disease, proving that an inherited immunodeficiency could be corrected by allogeneic hematopoietic cell transplantation (2). This success, published alongside a similar case in a patient with Wiskott–Aldrich syndrome (3), is universally recognized as the birth of curative transplantation for primary immunodeficiencies (4). Dr. Gatti’s contribution to this daring and compassionate experiment forever changed the prospects of children born with fatal immune diseases and contributed to launch the discipline of hematopoietic stem cell transplantation as a therapy for other nonmalignant diseases. He remained very proud that the transplanted patient was still living and thriving, a father of four, and he used to receive Christmas cards each year from members of the family. His research interests were shaped also at the Karolinska Institutet in Stockholm, where some of world’s most advanced research on cancer immunobiology was being performed in the laboratories of a prominent tumor immunologist, Dr. George Klein.

**Figure:**
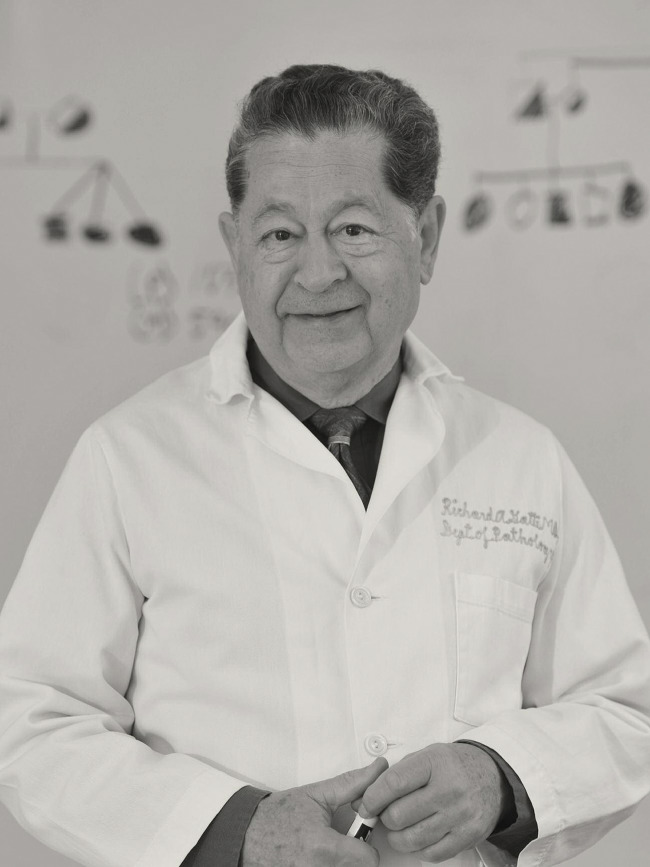
Richard A. Gatti. With permission from the 2012 UCLA Pathology and Laboratory Medicine Annual report (6).

After joining UCLA in 1974, Dr. Gatti turned his focus to ataxia-telangiectasia (A-T), a rare genetic disease combining neurodegeneration, immunodeficiency, and increase radiosensitivity, causing an increase susceptibility to various types of cancer, especially leukemia and lymphomas. He started to collect genetic pedigrees of ethnic groups in the U.S. and 20 other countries, and after a search lasting 7 years, he was finally able to map the gene for A-T in the chromosomal region of 11q23 (6). It took another 7 years and an international team of Britons, Israelis, and Americans to identify ATM as the causative gene of A-T at a time where there was no “human genome project map.” Dr. Gatti’s laboratory also contributed to mapping another closely related gene of DNA repair that causes Nijmegen breakage syndrome, a disease with radiosensitivity and high risk of cancer. He devoted most of his subsequent research to elucidating the role of the ATM gene and the mechanisms of DNA repair defects. He co-founded the UCLA Molecular Diagnostics Laboratory in 1987, integrating molecular genetics into patient care (5). For decades, his National Institutes of Health–funded projects and collaborations explored genotype–phenotype correlations, radiosensitivity, and innovative models such as induced pluripotent stem cells (iPSC)-derived “disease-in-a-dish” systems to test therapeutic avenues. He published over 300 papers, of which half are dedicated to the study of A-T. His work advanced not only fundamental knowledge but also the possibility of targeted therapies. In 2018, the global A-T community honored him with a Lifetime Achievement Award at the Ataxia-Telangiectasia Clinical Research Conference in Naples, Italy, praising his unwavering dedication to patients and families and his mentorship of younger investigators. Visiting his office at UCLA, it was typical to find piles of “pedigrees” of families with children suffering from A-T, with a wall covered with photos of children he helped diagnose and treat. For families facing rare genetic diseases, he was more than a researcher—he was a partner and advocate.

He was a multidimensional talent and unique contributor to the field. Colleagues enjoyed his scientific and medical insights, but also his bubbly humor and unshakable optimism. He was a terrific piano musician: he attended the prestigious Juilliard School in New York City in childhood, and already at the age of 15 he won competitions and played solo at radio stations in New York. He loved to entertain guests at his house and colleagues at conferences whenever there was a piano available. He is survived by his beloved wife, six children, and 12 grandchildren, who lovingly carry his spirit forward. He will be remembered as a pioneer and a mentor who gave his life to the service of science and patients.
